# Innovative parallel grasshopper optimization algorithm for reliability optimization

**DOI:** 10.1016/j.mex.2025.103759

**Published:** 2025-12-14

**Authors:** Dipti Singh, Neha Chand

**Affiliations:** Department of Applied Mathematics, Gautam Buddha University, Greater Noida, India

**Keywords:** Reliability optimization, Grasshopper optimization algorithm, Parallel approach, Constraint handling technique, Redundancy allocation problem

## Abstract

This study introduces a novel Parallel Grasshopper Optimization Algorithm (p-GOA), specifically designed to address reliability optimization problems. Although several hybrid algorithms exist in this field, the proposed p-GOA distinctly differs through its parallel cooperative strategy. Unlike sequential methods that apply techniques one after another, p-GOA simultaneously divides the population into two groups operating in parallel: one group employs a migration strategy (SOMA) for broad global exploration of the search space, while the other utilizes a mutation operator (NUMO) for focused local refinement of solutions. This dual-strategy parallel operation creates achieving a stronger balance between global exploration and local refinement, while a smart penalty-free method naturally steers the search toward workable solutions. When tested on four well-known reliability problems, the results demonstrate that our method consistently finds more reliable systems and converges faster than existing approaches, demonstrating its effectiveness in handling real-world engineering constraints.

● This study introduces a Parallel Grasshopper Optimization Algorithm (p-GOA) that integrates GOA, SOMA, and a Non-Uniform Mutation Operator (NUMO). It employs mutation, migration, and a parallel approach to efficiently explore both feasible and near-feasible regions without relying on penalty functions.

● The p-GOA dividing the population into two parallel groups—one updated using SOMA-based migration and the other using NUMO-based mutation. This dual-strategy, simultaneous processing not only accelerates convergence but also strengthens the balance between global search and local optimization.

● Specifically targets reliability optimization problems, particularly redundancy allocation issues where components must meet specific reliability and resource consumption (cost, weight, volume) constraints.

## Graphical abstract

**Figure:**
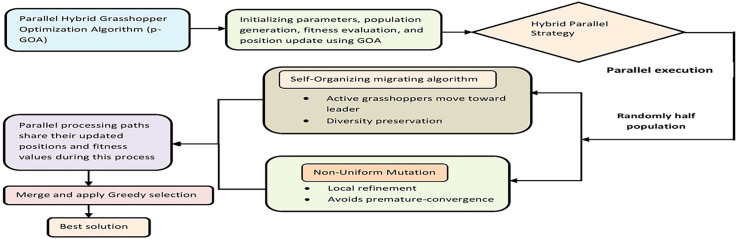

**Specifications table**

**Table:** 

**Subject area**	Mathematics and Statistics
**More specific subject area**	*Meta-heuristic Algorithm*
**Name of your method**	Hybridized meta-heuristic optimization
**Name and reference of original method**	Saremi, S., Mirjalili, S., & Lewis, A. (2017). Grasshopper optimisation algorithm: theory and application. *Advances in engineering software, 105*, 30–47.
**Resource availability**	The data used in this paper were generated through experimental runs.

## Background

Reliability-based design has become crucial in today’s society. The reliability redundancy allocation problem (RRAP) is a nondeterministic polynomial-time hard problem whose solution cannot be achieved using direct, indirect, or mixed search techniques [1]. As a result, researchers have resorted to a variety of methods, including meta-heuristic [[2], [3], [4]], and evolutionary algorithms [5], such as particle swarm optimization (PSO) [6,7], artificial immune system [8], cuckoo search algorithm [9], and biogeography-based optimizations (BBOs) [10]. Meta-heuristics have been widely chosen and effectively applied to address various challenges in reliability optimization, particularly in overcoming the computational complexity of RAP [11,12]. Over the years, researchers have investigated RAP, generally using either meta-heuristic optimization techniques or mathematical programming. In recent years, the development of meta-heuristic algorithms has become a primary research focus in this domain. Meta-heuristics often inspired by natural phenomena offer several advantages over traditional optimization methods, such as flexibility, global search capabilities. Several meta-heuristic approaches, including the Simulated Annealing Algorithm [13], Particle Swarm Optimization [14], and Cuckoo Search Algorithm [15], have been applied to the reliability allocation problem (RAP) in recent years. Aswin et *al.* solved the reliability optimization problems using the Jaya algorithm, and the results are validated through comparison with two other meta-heuristics [16]. Garg, H., proposed an effective optimization approach for solving reliability problems using biogeography-based optimization [10]. Similarly, Yeh et *al*. introduced an Artificial Bee Colony algorithm for solving RAP [17]. In recent years, many academicians have applied various meta-heuristics, including their improved and hybrid variants, to solve RRAP. To develop more efficient algorithms, new algorithms are now being developed by combining two or three algorithms. These are known as hybrid algorithms. For instance, Dos Santos et *al.* proposed a modified firefly algorithm integrated with chaotic sequences to optimize reliability redundancy [18]. Kanagaraj et *al*. developed a hybrid cuckoo search by merging it with a genetic algorithm (CS-GA) to solve the RAP [19]. Garg et *al*. presented a two-phase strategy using the artificial bee colony algorithm to address RAP [20]. Similarly, Deep and Dipti hybridized self-organizing migrating algorithms with genetic algorithms to tackle reliability optimization problems [21]. Marouani, H. solved the reliability optimization problem by improved particle swarm optimization (PSO) [22]. Bhandari et *al*. used a hybrid PSO–Grey Wolf Optimizer (HPSGWO) to solve RAP [23]. Garg et *al*. also proposed a hybrid genetic algorithm combined with particle swarm optimization to solve the redundancy allocation problem [24].

The development of a hybrid variant of GOA for redundancy allocation has been the main focus of current work. GOA is a novel optimization technique. The performance of four problems in the field of the reliability optimization model is examined in this paper, which draws inspiration from a variant of GOA and utilizes the advantages of GOA and SOMA and non-uniform mutation operators. The goal is to maximize system reliability while preserving the various resources and it has been noted that the new approach's results are all better than those already published in the literature.

## Method details

The proposed hybrid method uses three main components: the Grasshopper optimization algorithm, the self-organizing migrating algorithm, and a non-uniform mutation operator. These are combined to form a new variant for solving the reliability problem. The main features of each are explained below.

## Grasshopper optimization algorithm (GOA)

Inspired by the natural foraging and swarming behaviors of grasshoppers, Saremi et al. proposed the Grasshopper Optimization Algorithm (GOA), a novel and promising swarm intelligence technique [25]. Grasshoppers are well-known as destructive pests with a significant impact on agriculture and crop productivity. Their life cycle consists of two stages: larval and adult. During the larval stage, the swarm is characterized by slow movement and small steps. The swarming behavior of grasshoppers is mathematically represented by [26]

(1) $$X_{i}=S_{i}+G_{i}+A_{i}$$

In this case, X_i_ denotes the grasshopper's location, G_i_ denotes the force of gravity acting on it, S_i_ denotes social interaction, and A_i_ denotes wind advection. The random behaviour of Grasshopper is defined by following equation

(2) $$X_{i}=r_{1}S_{i}+r_{2}G_{i}+r_{3}A_{i}$$

where the random values r_1_, r_2_, and r_3_ range from 0 to 1.The values of social interaction S_i_ is defined as:

(3) Si=∑j=1j≠iNs(dij)Xj−Xidij

The distance between the i^th^ and j^th^ grasshoppers is defined as dij= |xj-xi|, the unit vector pointing from the i^th^ grasshopper to j^th^ grasshopper is given by $\hat{d_{ij}}$ =$\;\frac{x_{j}-x_{i}}{d_{ij}}$, where s denotes the strength of the social force function, defined as follows:

(4) $$s\left(r\right)\;=fe^{\frac{-r}{l}}\;-e^{-r}$$

Where the attractive length is denoted by l and the intensity of attraction by f

In Eq. (1) G_i_ calculated as:

(5) $$G_{i}=-g{\hat{e}}_{g}$$

Where g is a constant and $\hat{e_{g}}$ defined as unity vector toward the center of the earth.

The wind advection Ai is defined as:

(6) $$A_{i}=u\hat{e{\;}_{w}}$$

Where $\hat{e{\;}_{w}}$ is defined as unit vector in the direction of the wind and u denoted by drift constant.

Eq. (1) substituted the Si, Gi, and Ai values from Eqs. (3), 4, 5, and 6. After that, the grasshopper's updated position was calculated by:

(7) $$X_{i}=\sum_{\begin{array}{c} j=1\\ j\neq i \end{array}}^{N}s\left(\left|x_{j}-x_{i\;}\right|\right)\frac{x_{j}-x_{i}}{dij}-g\hat{e_{g}}+u{\hat{e}}_{w}$$

N denotes the total number of grasshoppers. The following is a calculation of the mathematical equation:

(8) $$X_{i}^{d}=c\;\sum_{\begin{array}{c} j=1\\ j\neq i \end{array}}^{N}c\frac{u_{bd}-l_{bd}}{2}s\left(\left|{x_{j}}^{d}-{x_{i}}^{d}\right|\right)\frac{x_{j}-x_{i}}{dij}+\hat{Td}$$

(9) $$c=cmax\;-t\frac{\left(C_{max}-C_{\text{min}}\right)}{T}$$

where the lower bound in the d^th^ dimension is denoted by l_bd_ and the upper bound by u_bd_. The best solution obtained so far in the d^th^ dimension space is indicated by the T_d_. G is equal to zero, and wind advection is always in the direction of the optimal solution T_d_, and S can be compared to the S component in Eq. (1). Where t is the current iteration, T is the maximum number of iterations, and c_max_ and c_min_ denote the maximum and minimum values of c, respectively.

Eq. (8) shows that a grasshopper’s next position is affected by its current location, the optimal solution position, and the position of all other grasshoppers. The first part of this equation represents the grasshopper’s current location relative to others in the swarm. The second term reflects the tendency of grasshoppers to move toward the food source. In Eq. (8), the two instances of the parameter c play different roles in determining the grasshopper’s next position. The second c is used to reduce the influence of the attraction, comfort, and repulsion zones, while the first c has the same effect as the inertia weight in Particle Swarm Optimization (PSO). A key feature of GOA is that it does not generate new solutions during the search process. Instead, it updates the position of each solution at every step by normalizing the distances between grasshoppers within a defined range [27]. Algorithm 1 provides the pseudo-code for the basic Grasshopper Optimization Algorithm.

**Algorithm 1:** pseudo-code for GOA.

Initialize grasshopper population X_i_, where i = 1, 2, …, n
Initialize parameters: c_max_, c_min_, MaxT, population size
Compare each grasshopper to identify which is the fittest
for t = 1 to MaxT do
Calculate c using Eq. (9)
for each grasshopper i do
Normalize the grasshopper's distance from one another
Use Eq. (8) to update the grasshopper's current location
Bring the current one grasshopper back if it jumps the boundaries.
end for
If a better solution is found then
Update t = t + 1
end If
end for
Return the optimal solution

## Self-Organizing migrating algorithm (SOMA)

A population-based stochastic optimization method based on the social behavior of a group of individuals is called the Self -Organizing Migrating Algorithm. Individuals in SOMA behave in a competitive-cooperative approach rather than in competition with one another. Only the locations of the individuals are changed throughout a generation, known as the "migration loop" (ML); SOMA does not create any new individuals during the search [28,29]. An active individual moves in the direction of the leader as follows:

(10) $$X_{i,j}^{MLnew}=X_{i,j,start}^{ML}+\left(X_{L,j}^{ML}-\;X_{i,j,start}^{ML}\right).t.\text{PRTVector}\;j.$$

where t ∈< 0, by step to, Path Length >, and ML is the migration loop. An individual new position is represented by$\;X_{i,j}^{\mathrm{MLnew}}$, an active individual position is defined by${\;X}_{i,j,\mathrm{strt}}^{\mathrm{ML}}$, and the position of leader is represented by${\;X}_{L,j}^{\mathrm{ML}}$**.** A parameter called the **PRT (perturbation) parameter** is used to introduce random variations in the search process. It plays a role in SOMA similar to that of mutation in Genetic Algorithms (GA). The value of this parameter lies between 0 and 1. Before an individual moves toward the leader, the PRT parameter is applied to generate a **PRT vector (perturbation vector).** For each coordinate of the individual, a random number is generated and compared with the PRT value to determine whether the coordinate will be perturbed.

$$if\;rnd_{j}<PRT$$

$$PRTVector_{j}=1;else$$

$$PRTVector_{j}=0;$$

endif

There are just two possible values for the randomly produced PRT vector: 0 and 1. The individual is not permitted to alter the position of an individual in the associated dimension if it is set to 0. Here, the dot (·) denotes element-wise multiplication, ensuring that only the dimensions allowed by the PRT vector are updated during movement toward the leader.

## Non-Uniform mutation operator (NUMO)

This chooses one solution, x_ij_, at random and sets its value in accordance with the following rule:

(11) $$x_{\text{ij}}^{\text{new}}=\left\{ \begin{array}{cc} x_{\text{ij}}+\left(ub_{j}-x_{\text{ij}}\right).\tau \left(t\right) & \text{if}\;a_{1}<0.5\\ x_{\text{ij}}-\left(x_{\text{ij}}+l_{\text{bj}}\right).\tau \left(t\right) & \text{if}\;a_{1}\geq 0.5 \end{array}\right.$$

where $\tau \left(t\right)={\left(a_{2}\left(1-\frac{t}{t_{max}}\right)\right)}^{b},$ a_1_ and a_2_ are two random numbers that lie between 0 and 1. a and b are constant parameters, t being the current generation, and t_max_ being the maximum number of generations. The bounds of the problem's parameters that need to be optimized are assumed to be (upper bound u_bj_ and lower bound l_bj_) [30]. Scalar multiplication is indicated by the dot operator (•) in Eq. (11). This equation uses scalar multiplication to update the chosen variable by multiplying random coefficients and parameters.

## Hybrid method

**Fig. 1:**
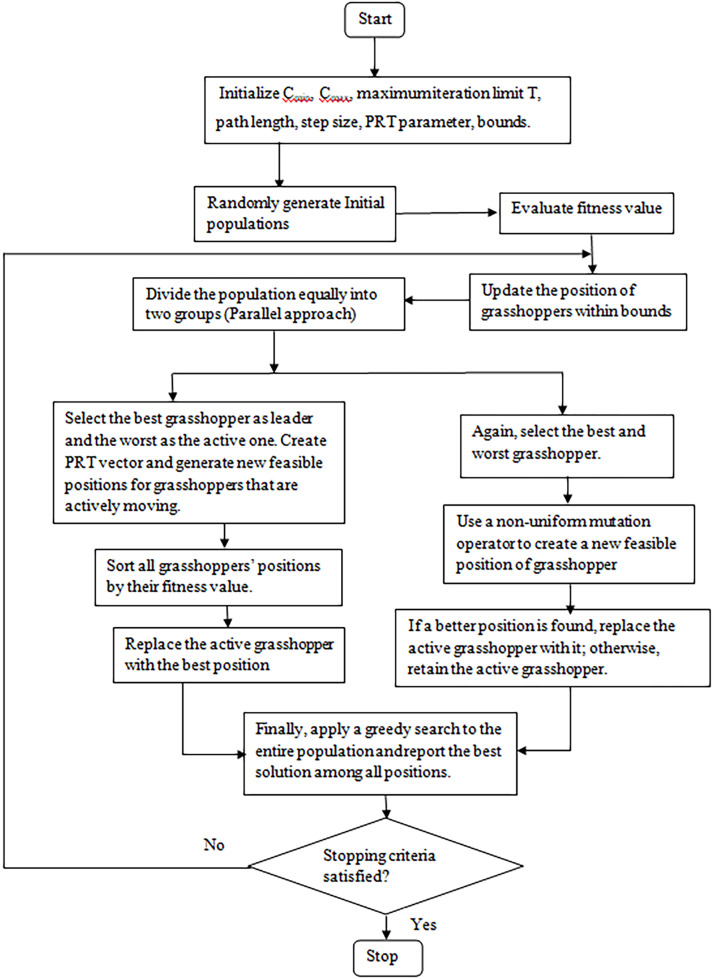
flowchart of proposed variant.

The objective of the proposed method is to solve reliability optimization problems using a hybrid variant of the Grasshopper Optimization Algorithm (GOA), coupled with SOMA and NUMO, and employing a penalty-free constraint-handling approach. Fig. 1 illustrates the workflow of the proposed parallel hybrid GOA model.

**1. Key Features of the Hybrid Parallel Grasshopper Optimization Algorithm (p-GOA)**

• **Parallel Implementation**: Reduces computational time and allows simultaneous calculation by dividing the population into two parallel groups. In the first group, half of the population updated by SOMA and the remaining half by NUMO.
• **Self-Organizing Migration**: Maintain diversity while guiding grasshoppers toward the global optimum.
• **Non-Uniform Mutation (NUMO)**: Improves local search and avoids premature convergence by continuously refining solutions.
• **Penalty-Free Constraint Handling:** Preserves the best feasible solutions and discourages infeasible solutions. Among infeasible solutions, those with smaller constraint violations are preferred.

In this method, no penalty is used for handling constraints. Instead, solutions that break fewer constraints are treated as feasible options.

**2. Constraint violation function** The constraint violation function can be expressed mathematically as follows:

(12) $$\psi \left(x\right)=\;\sum_{m=1}^{M}{\left[h_{m}\left(x\right)\right]}^{2}+\sum_{m=1}^{K}G_{k}{\left[g_{k}\left(x\right)\right]}^{2},$$

Where $h_{m}\left(x\right)$ represents the equality constraints and $g_{k}\left(x\right)$ represents the inequality constraints and G_k_ represents the Heaviside operator:

$$G_{k}=\left\{ \begin{array}{c} 0,\;g_{k}\left(x\right)\geq 0,\\ 1,\;g_{k}\left(x\right)<0. \end{array}\right.$$

For this, solutions within the feasible region have a value of constraint violation function zero. Conversely, solutions outside the feasible region have a non-zero constraint violation function value, indicating the degree to which the solution violates feasibility boundaries.

3. **Implementation of the Proposed p-GOA Method**

**3.1 Population initialization and position:** The initial population is randomly generated and evaluated using the defined fitness function. The best grasshopper is selected, and all positions are updated using Eq. (8). In the parallel structure, the population size is kept even so that half the individuals are updated using SOMA and the remaining half using NUMO.

**3.2 Parallel group division and update method:** Divide the equal number of grasshoppers randomly into two groups.

• **First group (SOMA-based update)**: In the SOMA group, the fittest grasshopper is chosen as the leader, while the least-fit grasshopper is selected as the active individual. “In each generation (migration loop), an active grasshopper moves in n steps of a predetermined length in the direction of the leader. Every step generates a new position for the active grasshopper. Eq. (10) describes how the active grasshopper moves toward the leader. The PRT (perturbation) parameter, which ranges from 0 to 1, randomly adjusts the movement direction of the active grasshopper toward the leader. The fitness value of this new population is sorted in ascending order. The constraint violation function is calculated at the best position of this sorted population. If ψ(x) = 0, the active grasshopper is changed with its current position and If ψ(x) > 0, the active grasshopper moves to the next best position in the sorted new population. In this way, the active grasshopper is updated with a new feasible solution position. If no feasible solution exists, the active grasshopper retains its previous position
• **Second group (NUMO-based mutation)**: In the second parallel group, the best and worst grasshoppers are selected. A grasshopper is selected at random, and a new grasshopper position is produced using the non-uniform mutation operator. This new solution is accepted if it outperforms the active grasshopper and satisfies the feasible conditions. This approach updates the active grasshopper with a new feasible position; if a feasible position is not found, the active grasshopper remains unchanged.
• After SOMA-based migration and NUMO-based mutation are completed, the parallel processing paths share their updated positions and fitness values during this step. The combined population then undergoes greedy selection to form the next generation. No intermediate communication is required

**3.4 Selection of good quality solutions**: Apply the greedy search on all grasshoppers and repeat the process until the termination requirement is satisfied.

Algorithm 2 provides the pseudo-code of proposed method.

**Algorithm 2:** Hybrid parallel grasshopper optimization algorithm.

Initialize parameters: c_max_, c_min_, MaxT,path length, step size, PRT parameter, and bounds
Generate initial population of grasshoppers X_i_, where i = 1, 2, …, N
Evaluate fitness value for each grasshopper
for t = 1 to MaxT do
Update positions of all grasshoppers using GOA within bounds
Evaluate fitness of all grasshoppers
Divide the population into two equal groups: Group 1 and Group 2
{— Operations on Group 1: SOMA based Update —}
Select the best and worst grasshopper in Group 1
Create PRT vector
Generate new feasible positions for actively moving grasshoppers using Eq. (10)
Evaluate and sort Group 1 by fitness
If improved position found then
Replace the active grasshopper with the best one in Group 1
end if
{— Operations on Group 2: Non-uniform Mutation —}
Select the best and worst grasshopper in Group 2
Apply non-uniform mutation operator to generate a new feasible position
If the new position is better then
Replace the active grasshopper with the new position
else
Retain the original active grasshopper
end If
Apply greedy search to the entire population
Record and retain the best solution found so far
end for
Return the best solution found

4. **Computational steps:** The computational steps are as follows:

1. Randomly generate the initial populations and evaluate fitness solutions.
2. Calculate c using Eq. (9).
3. Normalize the distance between grasshoppers.
4. Update the grasshopper's current location using Eq. (8).
5. Bring the grasshopper back to the nearest boundary if its updated position exceeds it.
6. Now divide the population into two equal groups for parallel updates.

**First group (SOMA-based update)**

• Select the best-fit grasshopper as leader and the worst will be an active one.
• Generate the PRT vector and update the locations for grasshoppers that are actively moving using Eq. (10).
• Sort all grasshoppers’ positions according to their fitness value and check the feasibility condition for the best grasshopper in the sorted population.
• If feasibility criteria are satisfied replace the active grasshopper with the best position if active grasshopper is worse than the best position else move to the next best position in the sorted order.

**Second Group (NUMO based mutation)**

• Select the best and worst grasshopper in this group.
• Randomly choose a grasshopper and apply the non-uniform mutation operator using Eq. (11) to generate a new position.
• Accept the new position only if it improves fitness and satisfies the constraint condition, replacing the active grasshopper.

7. Apply greedy search to the entire population.

8. This process is repeated until certain termination requirements are met.

9. Finally, the optimal solution is reported.

## Method validation

The proposed method was validated using well-established reliability optimization problems.

**1. Problem formulation: reliability redundancy allocation problems**

RAPs are typically formulated as integer or mixed-integer nonlinear optimization problems due to the discrete nature of component selection and the nonlinear relationship between system reliability and its constituent elements [14]. The general form of the reliability-redundancy allocation problem is

$$Maximize\;R_{s}\left(r_{1},r_{2},\ldots \ldots ..r_{m},x_{1},x_{2},\ldots \ldots ,x_{m}\right)$$

$$\text{Subject}\;\text{to}\;g\left(r_{1},r_{2},\ldots \ldots ..r_{m},x_{1},x_{2},\ldots \ldots ,x_{m}\right)\leq b$$

$$0\leq r_{i}\leq 1;r_{i}\epsilon \left[0,1\right]\subset R;i=1,2,\ldots \ldots .,m$$

$$1\leq x_{i}\leq x_{i,\text{max}};x_{i}\epsilon Z^{+}$$

where g(·) represents the set of constraints normally related to the weight, volume, and cost of the system; r_i_ and x_i_ are the reliability and number of redundant components in the subsystem i^th^, respectively; m is the number of subsystems in the system; and b is the vector of resource limitations. Also, Rs(·) shows the objective function of overall system reliability. Certain variables, such as the number of redundancies (x_i_), are positive integers, whereas other variables, such as component reliability (r_i_), are real numbers between 0 and 1. This makes the problem a mixed-integer programming problem. To maximize the total system reliability, the primary objective of this problem is determining the number of components (x_i_ and r_i_) in each subsystem.

**2. Notations m Number of subsystems in the system**

M Number of constraints x_i_ The number of components in subsystem. x (x_i_,x_2,…..,_x_n_), the vector of redundancy allocation for the system. r_i_ Reliability of each components in subsystem. r (r_1_, r,r_m_), the vector of component reliabilities for the system. g_j_ The j^th^ constraint function. w_i_ The weight of each component in subsystem. c_i_ The cost of the each component in subsystem. v_i_ The volume of each component in subsystem**.**

R_i_ 1 − (1 − ri^)ni^ is the reliability of the i^th^ subsystem.

Q_i_ 1 − Ri is the unreliability of the i^th^ subsystem. x_i_,_max_ Maximum number of components in subsystem.

Rs The system reliability.

C, W, V The upper limit of the system’s cost, weight and volume respectively.

Four case studies are taken into consideration in order to assess how well the suggested method performs on reliability redundancy allocation challenges. These include large-scale systems, series systems, series–parallel systems, complicated (bridge) systems, and over speed protection systems.

**3. Four reliability optimization problems**

3.1 A series system

**Fig. 2:**
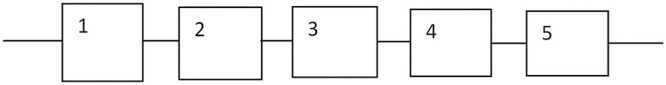
Series system.

A nonlinear mixed-integer programming issue for a series system with five subsystems is the subject of this case study as seen in Fig. 2 [10].The following is the formulation of the problem.

$$Maximize.\;R_{s}\left(r,x\right)\;=\prod_{i=1}^{5}\left[1-{\left(1-r_{i}\right)}^{x_{i}}\right]$$

$$\text{Subject}\;\text{to}:g_{1}\left(r,x\right)\;=\;\sum_{i=1}^{5}v_{i}x_{i}^{2}\leq V\left(a\right)$$

$$g_{2}\left(r,x\right)\;=\;\sum_{i=1}^{5}\alpha_{i}.{\left(-1000/lnr_{i}\right)}^{\beta_{i}}.\left[x_{i}+\text{exp}\left(x_{i}/4\right)\right]\leq C\left(b\right)$$

$$g_{3}\left(r,x\right)\;=\;\sum_{i=1}^{5}w_{i}.x_{i}\left[x_{i}.\exp\left(x_{i}/4\right)\right]\leq W\left(c\right)$$

$$0.5\leq r_{i}\leq 1,r_{i}\epsilon \left[0,1\right]\subset R^{+},1\leq x_{i}\leq 5,x_{i}\epsilon Z^{+};i=1,2,\ldots .,5$$

3.2 A series–parallel system

**Fig. 3:**
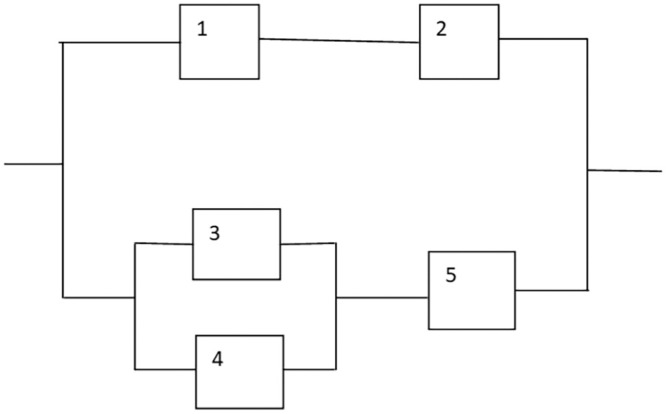
Series –parallel system.

As seen in Fig. 3 [10], this case study involves nonlinear mixed-integer programming for a series–parallel system with five subsystems.

$$Maximize.\;R_{s}\left(r,x\right)\;=\;1-\left(1-R_{1}R_{2}\right)\left[1-\left(R_{3}+R_{4}-R_{3}R_{4}\right)R_{5}\right]$$

$$\text{Subject}\;\text{to}:\;g_{1}\left(r,x\right),\;g_{2}\left(r,x\right),g_{3}\left(r,x\right)\left(\text{as}\;\text{specified}\;\text{by}\;\left(a\right),\;\left(b\right),\;\left(c\right)\right)$$

$$0.5\leq r_{i}\leq 1,\;r_{i}\epsilon \left[0,1\right]\subset R^{+},1\leq x_{i}\leq 5,x_{i}\epsilon Z^{+};i=1,2,\ldots .,5$$

$$\text{where}R_{i}=1-{\left(1-r_{i}\right)}^{xi}$$

3.3 A complex (bridge) system

**Fig. 4:**
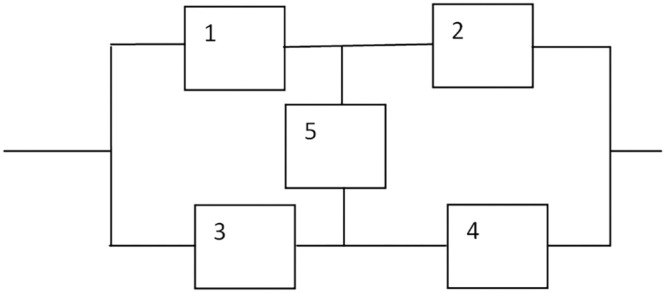
Complex (bridge) system.

The nonlinear mixed-integer programming issue for a complex (bridge) system with five subsystems is the subject of this case study as illustrated in Fig. 4[10]. The following is the formulation of the problem:

$$Maximize.\;R_{s}=\;R_{1}R_{2}+R_{3}R_{4}+R_{1}R_{4}R_{5}+R_{2}R_{3}R_{5}-R_{1}R_{2}R_{3}R_{4}-R_{1}R_{2}R_{3}R_{5}-R_{1}R_{2}R_{4}R_{5}-R_{1}R_{3}R_{4}R_{5}-R_{2}R_{3}R_{4}R_{5}+2R_{1}R_{2}R_{3}R_{4}R_{5}$$

$$\text{Subject}\;\text{to}:g_{1}\left(r,x\right),\;g_{2}\left(r,x\right),\;g_{3}\left(r,x\right)\;\left(\text{as}\;\text{specified}\;\text{by}\;\left(a\right),\;\left(b\right),\;\left(c\right)\right)$$

$$0.5\leq r_{i}\leq 1,\;r_{i}\epsilon \left[0,1\right]\subset R^{+},1\leq x_{i}\leq 5,x_{i}\epsilon Z^{+};i=1,2,\ldots .,5$$

$$\text{where}R_{i}=1-{\left(1-r_{i}\right)}^{xi}$$

3.4 An over speed system for a gas turbine

**Fig. 5:**
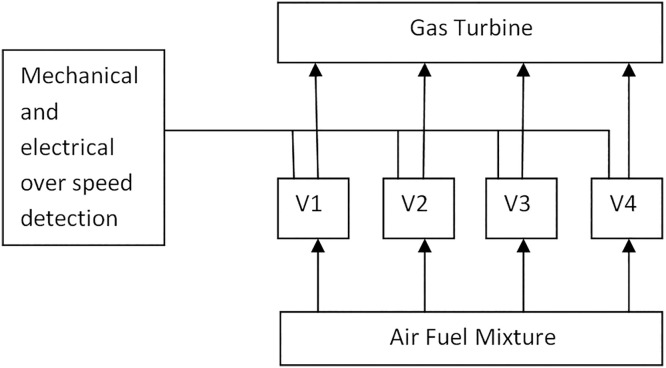
over speed protection system.

The mechanical and electrical systems continuously give over speed detection. The fuel supply must be cut off when an over speed occurs. Four control valves (V_1_–V_4_) need to be closed for this reason. Fig. 5 [10] illustrates how the control system is modeled as a four-stage series system. Finding the ideal level of r_i_ and x_i_ at each stage I in order to maximize system reliability is the objective. The following is the formulation of this case study:

$$Maximize\;R_{s}\left(r,x\right)\;=\prod_{i=1}^{4}\left[1-{\left(1-r_{i}\right)}^{x_{i}}\right]$$

$$\text{Subject}\;\text{to}:g_{1}\left(r,x\right)=\sum_{i=1}^{4}v_{i}x_{i}^{2}\leq V$$

$$g_{2}\left(r,x\right)=\sum_{i=1}^{4}\alpha_{i}.{\left(-1000/lnr_{i}\right)}^{\beta_{i}}\left[x_{i}+\text{exp}\left(x_{i}/4\right)\right]\leq C$$

$$g_{3}\left(r,x\right)\;=\;\sum_{i=1}^{4}w_{i}.x_{i}\left[x_{i}.\exp\left(x_{i}/4\right)\right]\leq W$$

$$0.5\leq r_{i}\leq 1,\;r_{i}\epsilon \left[0,1\right]\subset R^{+},1\leq x_{i}\leq 10,x_{i}\epsilon Z^{+};i=1,2,\ldots .,4$$

Four test problems are taken into consideration in order to assess how well the suggested method performs in resolving the mixed-integer non-linear reliability design issues. The mixed-integer nonlinear programming problem can describe all of these issues involving maximizing the systems' reliability under several nonlinear constraints. Redundancy allocations and component reliabilities must be determined dynamically for each problem. Table 1 shows the controlling parameter for the suggested approach. The input parameters for four problems are shown in Tables 2, 3, and 4. The best results obtained for these problems are presented in Table 5, Table 6, Table 7, Table 8 and are compared with previous studies’ published results [19,20,[31], [32], [33]]. From the results it can be interpreted that the proposed approach outperforms other alternative approaches taken into consideration and is efficient to solve such kinds of problems. Table 5 summarizes the results for problem 1, which shows that the best solution obtained by the proposed method is 0.931682, which is comparable to the solutions produced by the other methods found in the literature. The experiment's findings for problem 2 are displayed in Table 6 and show that the best optimal solution provided by the suggested method (Rs = 0.999976649049) is significantly superior to the solutions provided by [19,20,[31], [32], [33]]. Table 7, Table 8 show that the optimal solution found through the suggested method is superior to the existing solution given by [19,20,[31], [32], [33], [34]]. An analysis has been repeated for 20 independent runs to remove the stochastic discrepancy in order to statistically compare the performance of the suggested algorithm with other meta-heuristic algorithms. Table 9 presents statistical analysis of four optimization problems using the proposed method based on 20 independent runs; showing the best, worst, and mean fitness values and standard deviations. The p-GOA algorithm was implemented in Python 3.13.2 and executed on an Intel Core i3–1005G1 processor with 4 GB RAM. Across 20 independent runs of 1500 iterations each, the average computation times were Series System (15.2 s), Series-Parallel System (16.0 s), Complex Bridge System (28.4 s), and Over speed Protection System (16.0 s).

**Table 1:** Control parameter for the proposed p-GOA.

Control parameter	Fine tuned value
Population size	20 for problem 1,2,3 and 30 for problem 4
Maximum iteration	1500 iteration
PRT	0.1 and 0.3
Step size	0.05
Path length	2

**Table 2:** input parameter of the series and complex system.

subsystem	10^5^α_i_	β_i_	v_i_	w_i_	V	C	W
1	2.330		1	7			
2	1.450		2	8			
3	0.541	1.5	3	8	110	175	200
4	8.050		4	6			
5	1.950		2	9

**Table 3:** input parameter of the series- parallel system.

subsystem	10^5^α_i_	β_i_	v_i_	w_i_	V	C	W
1	2.500		2	3.5			
2	1.450		4	4			
3	0.541	1.5	5	4	180	175	100
4	0.541		8	3.5			
5	2.100		4	3.5

**Table 4:** input parameter of the over speed protection system.

subsystem	v_i_	w_i_	V	C	W
1	1	6			
2	2	6	250	400	500
3	3	8			
4	2	7

**Table 5:** optimal solution for problem 1.

	Valiant et.al [31]	Kanagaraj et al [19].	Garg et al [20]	Liu [32]	Marouani, H. et al [33]	This study
R_sys_	0.931682387	0.931682388	0.931682388	0.931682388	0.93168138	0.931682
X_1_-X_5_	(3, 2, 2, 3, 3)	(3, 2, 2, 3, 3)	(3, 2, 2, 3, 3)	(3, 2, 2, 3, 3)	(3, 2, 2, 3, 3)	(3, 2, 2, 3, 3)
r_1_	0.779416938	0.779398871	0.779403565	0.779398875	0.779760	0.779607
r_2_	0.871833278	0.871837021	0.871833201	0.871837014	0.872088	0.871988
r_3_	0.902885082	0.902885355	0.902886412	0.902885361	0.902689	0.902779
r_4_	0.711393868	0.711402515	0.787808549	0.787799488	0.711405	0.711380
r_5_	0.78779488	0.787799488	0.787808549	0.787799488	0.787208	0.787909

**Table 6:** optimal solution for problem 2.

	Valiant et.al [31]	Kanagaraj et al [19].	Garg et al [20]	Liu [32]	Marouani, H. et al [33]	This study
R_sys_	0.999976649	0.99997665	0.999976649	0.99997665	0.99997661	0.9999860
X_1_-X_5_	(2, 2, 2, 2, 4)	(2, 2, 2, 2, 4)	(2, 2, 2, 2, 4)	(2, 2, 2, 2, 4)	(2, 2, 2, 2, 4)	(3, 2, 2, 2, 4)
r_1_	0.819927087	0.819660256	0.819737753	0.819659319	0.822012	0.78700
r_2_	0.884526766	0.844981615	0.8449911	0.844980736	0.843656	0.8771270
r_3_	0.895491554	0.895519305	0.895552954	0.895506418	0.891290	0.889270
r_4_	0.895440692	0.895492245	0.895433687	0.895506432	0.898698	0.8900970
r_5_	0.868318775	0.868447587	0.868434482	0.868447751	0.868249	0.8574880

**Table 7:** optimal solution for problem 3.

	Valiant et.al [31]	Kanagaraj et al [19].	Garg et al [20]	Liu [32]	Marouani, H. et al [33]	This study
R_sys_	0.99988964	0.99988964	0.9998896	0.99988964	0.99988963	0.999889
X_1_-X_5_	(3, 3, 2, 4, 1)	(3, 3, 2, 4, 1)	(3, 3, 2, 4, 1)	(3, 3, 2, 4, 1)	(3, 3, 3, 3, 1)	(3, 3, 2, 4, 1)
r_1_	0.828094038	0.82808567	0.827970276	0.828086469	0.827642	0.828157
r_2_	0.858004485	0.85780605	0.857874756	0.85780477	0.857478	0.858089
r_3_	0.914162924	0.91424006	0.914186402	0.914240669	0.914196	0.914386
r_4_	0.647907792	0.64814375	0.895433687	0.895506432	0.649273	0.646789
r_5_	0.704565982	0.70418228	0.703575311	0.704162105	0.704092	0.709239

**Table 8:** optimal solution for problem 4.

	Wu et al [34]	Valiant et.al [31]	Kanagaraj et al [19]	Garg et al [20]	Marouani, H. et al [33]	This study
R_sys_	0.99995467	0.99995468	0.999954675	0.999954675	0.99995467	0.9999550
X_1_-X_5_	(5, 6, 4, 5)	(5, 5, 4, 6)	(5, 5, 4, 6)	(5, 5, 4, 6)	(5, 6, 4, 5)	(5, 5, 4, 6)
r_1_	0.90163164	0.901614595	0.901613407	0.90162681	0.901489	0.901486
r_2_	0.8499702	0.888223369	0.888223375	0.888208356	0.850035	0.888074
r_3_	0.94821828	0.948141029	0.94814211	0.948134378	0.948129	0.948075
r_4_	0.88812885	0.84992089	0.849920787	0.849942136	0.888238	0.850385

**Table 9:** Statistical analysis of four problems based on 20 runs.

Problem	Best	Worst	Mean	Standard Deviation(×$10^{-6})$
1	0.9316820	0.9316760	0.9316790	1.50
2	0.9999860	0.9999781	0.9999821	2.20
3	0.9998890	0.9998860	0.9998875	0.75
4	0.9999550	0.9999548	0.9999549	5.10

The cost and reliability of four complex systems have been optimized in this study using a parallel Grasshopper Optimization Algorithm (p-GOA). These problems fall within the domain of reliability engineering. In these optimization tasks, both the redundancy level (i.e., the number of redundant components) and the associated reliability of each component within each subsystem are determined simultaneously under various constraints. The p-GOA yields superior solutions in three of the four problems when compared to previously reported results, while achieving comparable outcomes in the fourth case. A population size of 20 was used to solve the first three problems, and 30 for the fourth. Based on the results of this study, it is concluded that p-GOA is a practical and effective method for addressing reliability optimization problems. Future research will aim to extend p-GOA to solve more complex reliability optimization problems and to integrate machine learning techniques. This integration will not only improve the speed and performance of GOA but also enable it to address large-scale benchmark problems and system reliability allocation tasks involving a greater number of variables.

## Limitations

The effectiveness of our proposed p-GOA method should be considered alongside its limitations:

1. **Scalability**: The algorithm was validated on four standard test problems. Its efficiency when applied to massively large-scale reliability systems needs further investigation.
2. **Parameter Tuning**: Like many meta-heuristics, p-GOA relies on control parameters that were carefully tuned for this study. Applying it to new problems may require a similar tuning effort.
3. **Computational Overhead**: The parallel structure, while accelerating convergence, increases the computational cost per iteration compared to simpler algorithms.

## Ethics statements

This research did not involve human subjects, animal experiments, or data collected from social media platforms. No ethical approval was required.

## CRediT authorship contribution statement

**Dipti Singh:** Supervision, Validation, Writing – review & editing. **Neha Chand:** Conceptualization, Methodology, Formal analysis, Writing – original draft, Resources.

## Declaration of competing interest

The authors declare that they have no known competing financial interests or personal relationships that could have appeared to influence the work reported in this paper.

## Data Availability

The data used in this paper were generated through experimental runs.
